# Distribution patterns of aquatic birds in a high-Andean wetland in southeastern Peru: An approach based on environmental factors

**DOI:** 10.1371/journal.pone.0320987

**Published:** 2026-03-26

**Authors:** Carlos Lazo, Renny Daniel Diaz, Alexis Díaz, Victor Bustinza, Wilfredo Chavez, Aracely Dayana Machaca, Yoana Yarasca, Edwin Calderon, Maria Masias

**Affiliations:** 1 Oficina Desconcentrada Macro Región Sur, Instituto Nacional de Investigación en Glaciares y Ecosistemas de Montaña, Cusco, Perú; 2 Círculo de Investigación de Ornitología, Universidad Nacional Agraria La Molina, Lima, Perú; 3 Centro de Investigación Vertebrate, Universidad Nacional San Antonio Abad del Cusco, Cusco, Perú; 4 División de Ornitología, Centro de Ornitología y Biodiversidad, Lima, Perú; King Fahd University of Petroleum & Minerals, SAUDI ARABIA

## Abstract

High-Andean wetlands play a crucial role in avian biodiversity conservation, serving as ecological oases in arid mountain regions. These ecosystems face increasing anthropogenic pressures, yet research remains limited, hindering conservation efforts. We investigated aquatic bird distribution patterns in Lake Piuray (southeastern Peruvian Andes) and their relationship with environmental variables like water depth and chlorophyll-a content. From December 2022 to November 2023, we conducted monthly surveys at 13 points across four lake zones representing natural and altered habitats. Using interpolation and additive models, we analyzed relationships between aquatic bird abundance and environmental factors, comparing seasonal and zonal differences. We recorded 44 aquatic bird species (19,768 individuals). Areas with depths < 1 m and intermediate-to-high chlorophyll-a levels (I543 index: 0.20–0.25) showed highest abundance and richness. Lakeshore beaches supported greater aquatic bird concentrations than deeper zones. At the family level, shorebirds (Charadriidae and Recurvirostridae) preferred shallow waters, while diving birds (Anatidae and Podicipedidae) tolerated greater depths. Although community-level metrics showed no seasonal differences, six families exhibited abundance variations between wet and dry seasons: Phalacrocoracidae, Scolopacidae, and Recurvirostridae were more abundant in wet seasons, while Charadriidae, Laridae, and Phoenicopteridae peaked in dry seasons. These results highlight the importance of shallow areas with high chlorophyll-a concentrations for the conservation of aquatic birds in high-Andean wetlands. This study is one of the few to analyze the influence of environmental and temporal factors on the distribution of high-Andean aquatic birds, and its representative nature makes it a valuable model for identifying priority areas for the conservation of these species.

## Introduction

The tropical Andes are one of the most biodiverse regions globally and represent a conservation priority [1]. However, these ecosystems face constant anthropogenic pressures (agriculture, mining, and overgrazing), particularly due to changes in land use and land cover [2,3]. Within this threatened landscape, high-altitude wetlands (≥3000 m asl) emerge as critically important conservation areas, as they are the primary providers of ecosystem services due to their high primary productivity and water regulation functions [4]. Additionally, these wetlands act as ecological “oases” because of their significant differences in productivity and biodiversity compared to the surrounding areas, especially during the dry season [5,6]. However, significant knowledge gaps remain regarding the extent, characterization, and biological richness of these wetlands in South America [7].

Aquatic birds are widely recognized as effective bioindicators of wetland health due to their ecological specialization and sensitivity to habitat alterations [8,9]. These species respond rapidly to environmental changes such as water level fluctuations, nutrient enrichment, habitat fragmentation, and human disturbances [10]. Their responsiveness is linked to the diverse morphological, physiological, and behavioral adaptations they have evolved to exploit different habitats based on specific environmental variables [11]. For example, greater coverage of emergent vegetation enhances waterbird richness and abundance, particularly during the breeding season, by providing suitable nesting sites [12,13]. Similarly, water depth correlates positively with tarsus length in shorebirds and neck length in diving ducks [14,15]. Wetland size is also a key factor, as habitat configuration influences species preferences; larger wetlands tend to support greater environmental heterogeneity, thereby sustaining higher bird diversity [16,17]. Understanding these factors is critical for interpreting habitat selection processes and their implications for aquatic birds’ spatial distribution [18,19].

In South America, the wide altitudinal variation, ranging from the lowlands of the Amazon and the Pacific coast to the high Andes, generates a diversity of wetlands with contrasting environmental conditions [20]. This heterogeneity significantly influences the ecological responses of aquatic birds, whose communities vary according to the predominant environmental factors in each region. In Amazonian wetlands, species richness is positively associated with water depth and isolation of water bodies, and negatively correlated with water transparency [21]. Along the Pacific coast, factors such as shoreline length, emergent vegetation cover and water-level fluctuations determine resting and feeding patterns as well as species richness [22]. Similarly, in subtropical plains, features like high salinity, muddy shores, and seasonal lagoon dynamics determine the presence of specialized species such as plovers and flamingos [19]. The high-altitude Andean wetlands present particular ecological conditions marked by high seasonal and spatial variability in water availability. In the northern Andes, during the dry season, a decrease in bird abundance has been documented and attributed to reduced resource availability [23]. Furthermore, intermediate eutrophication levels have been observed to favor the richness and abundance of herbivorous species, while high levels tend to disproportionately benefit the Slate-colored Coot (*Fulica ardesiaca*), a widely distributed species throughout the Andes [24].

Despite these advances, knowledge about the influence of environmental factors on waterbird distribution along the Andes remains limited [5,25]. In Peru, this gap is particularly evident as most research has focused on coastal wetlands including mangroves, estuaries, and littoral lagoons [26], despite the country having nearly equal proportions of coastal (46.6%) and Andean (47.2%) wetlands [27]. This disparity underscores the urgent need to expand studies of high Andean wetlands and the factors determining their waterbird community distribution. Lake Piuray in southeastern Peru represents a notable example of an Andean lake facing growing ecological challenges. This freshwater system plays a crucial role in regional water supply while supporting significant bird diversity: the lake and its basin host at least 147 bird species including migratory and endemic populations, with several threatened taxa [28]. However, Lake Piuray faces increasing anthropogenic pressures from urban expansion, agricultural intensification, and tourism development, all contributing to habitat fragmentation, pollution, and hydrological alterations [29]. Evidence suggests a progressive decline in avian diversity since 1993 [30], though systematic assessments remain limited, emphasizing the need for targeted research.

The objectives of this study were to (1) characterize spatiotemporal distribution patterns of aquatic birds in Lake Piuray and (2) identify associated environmental variables, particularly water depth and primary production. We evaluated species richness, abundance and diversity at community and family levels across four lake zones during both wet and dry seasons. These findings provide insights applicable to wetlands throughout Peru's central-southern Andes and other rapidly changing mountain regions worldwide.

## Methods

### Study area

Lake Piuray (13°25’0.00"S, 72°1’58.00"W; 2669 m asl) is located in the district of Chinchero, province of Urubamba, department of Cusco, Peru. This lake covers an area of 356 ha and reaches a maximum depth of 43.85 m. It is considered one of the main water sources for the city of Cusco, supplying 38% of its population [31]. The lake receives its water recharge primarily from the surrounding ecosystems of humid puna grasslands and Andean shrublands located in the headwaters of the Piuray micro-watershed [32]. The humid puna grasslands are dominated by low-growing grass turf and scattered bunchgrasses reaching maximum heights of 1.5 meters, while the Andean shrublands feature woody shrub vegetation not exceeding 4 meters in height [27,28]. The lakeshore areas comprise agricultural zones primarily dedicated to potato and corn cultivation, eucalyptus forest plantations, and small urban settlements. Regarding aquatic vegetation, some lake edge sectors show emergent vegetation predominantly from totora reed marshes, while other zones feature muddy lacustrine beaches characterized by sparse vegetation cover and extensive shallow littoral areas. In 2023, the area experienced an average annual precipitation of 809.6 mm, with two well-defined climatic seasons: a wet season (November – April) with 691.8 mm and a dry season (May – October) with 117.8 mm.

### Selection of sampling sites

**Fig 1:**
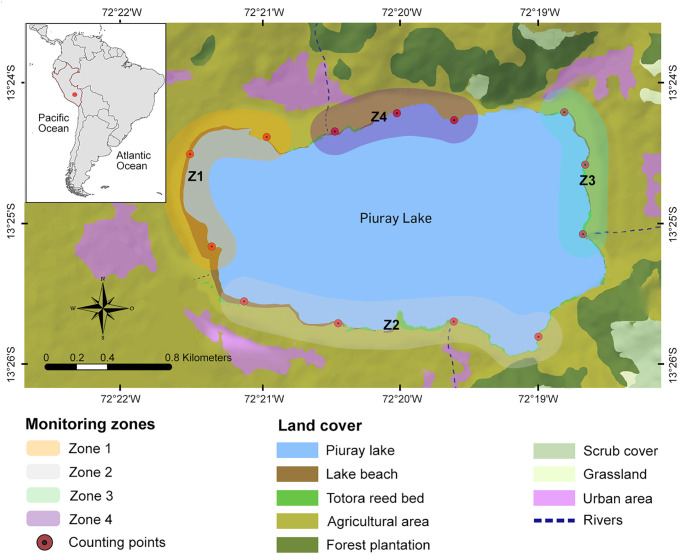
Location of study area and counting points on the land cover map of Lake Piuray and nearby areas. Base map generated with Landsat 8 imagery (Product ID: LC08_L1TP_004069_20210703_20210712_02_T1), courtesy of the U**.**S. Geological Survey (USGS) Earth Resources Observation and Science (EROS) Center. Public domain data available via EarthExplorer: https://earthexplorer.usgs.gov/. Data provided under the Creative Commons Zero (CC0 1.0) public domain dedication: https://www.usgs.gov/data-management/data-licensing.

To facilitate the evaluation of the area and capture the specific characteristics of each region of the wetland, the study area was divided into four zones (Fig 1) covering the entire perimeter of Lake Piuray [33]. Zone 1 (Z1), located to the east, includes extensive agricultural areas and shallow lakeshore beaches. Zone 2 (Z2), situated to the south, also encompasses agricultural lands and a narrow strip of shallow lakeshore beach; however, the eastern end is dominated by considerably deep waters. Zone 3 (Z3), to the west, is characterized by extensive agricultural areas that extend almost to the shoreline, leaving little space for lakeshore beaches; here, nearshore waters are shallow but increase rapidly in depth toward the center of the lake. Finally, Zone 4 (Z4), to the north, is characterized by its proximity to eucalyptus plantations and agricultural areas located at the edge of the lake, with almost no lakeshore beach and deep waters.

A total of 13 permanent counting points with a radius of 150 m were established [34]. Each of the four zones had three counting points, except for Zone 2, which had four due to its larger size. The average distance between points was 562.84 m (± 136.24 m standard deviation). Each counting point was located at the edge of the lake, surrounding it, in areas that facilitated the observation and identification of aquatic birds [35,36].

### Aquatic bird censuses

Between December 2022 and November 2023, monthly aquatic bird censuses were conducted by four observer teams, one assigned to each study zone. The censuses were carried out simultaneously to avoid double counting, following the protocol described by Ministerio de Ambiente del Peru (MINAM) [35]. Each census began between 07:00 and 08:00 h and concluded between 10:00 and 11:00 h (PET). At each counting point, all aquatic birds seen or heard were recorded during a 15-minute survey, with two consecutive repetitions within a 150 m radius. A minimum distance of 15 meters between observers and aquatic birds was maintained to minimize disturbance.

Three Nikon P950 cameras and one Nikon D5600 equipped with a Sigma 150–600 mm lens were used, along with eight Vortex 8x42 binoculars. These devices were distributed among the four observer groups, ensuring that each team had two binoculars and one camera. Species identification was based on the prior experience of the observers; in more challenging cases, the field guide Birds of Peru [37] and the Merlin Bird ID mobile application [38] were consulted. Data were recorded in field notebooks. No permit from the Servicio Nacional Forestal y de Fauna Silvestre (SERFOR) was required, as no individuals were handled or collected.

### Data analysis

A total of 11 families of aquatic birds were recorded across both seasons, with 35 species observed during the rainy season and 39 during the dry season (S1 File). The analysis was conducted at the family level because several species of interest, including the Andean ibis (*Theristicus branickii*), the cinnamon sandpiper (*Calidris subruficollis*), and the Greater Yellowlegs (*Tringa melanoleuca*) listed as Near Threatened and the lesser yellowlegs (*Tringa flavipes*) listed as Vulnerable by the International Union for Conservation of Nature [39], as well as 16 boreal migratory species from the families Scolopacidae (12), Charadriidae (2), Laridae (1), and Anatidae (1), were recorded only occasionally during the study period (S1 File). Given the low number of records, analysis at the species level was not practical. Aggregating data at the family level enabled a more reliable assessment of spatial patterns and their relationship with environmental variables. This approach has been effective in other studies of aquatic birds, helping to detect shared ecological responses among species with similar distributions [40,41].

### Determination of abundance patterns in the aquatic bird community and families

Two complementary analyses were performed: one at the community level and another at the aquatic bird family level. To visualize spatial abundance patterns in Lake Piuray and identify priority conservation areas, spatially continuous abundance distribution maps were generated using RStudio [42]. A Generalized Additive Model (GAM) was used, following the methodology proposed by Wood [43], to correlate the abundance of the community and each aquatic bird family (response variable) with chlorophyll-a content and lake depth (explanatory variables). A non-parametric model was chosen due to the expected nonlinear relationships between environmental variables and aquatic bird abundance [11].

To estimate the spatial distribution of aquatic birds in the study area, the Inverse Distance Weighting (IDW) interpolation method was applied. This method estimates abundance values in unsampled areas based on data from counting points, converting point data into continuous raster data for easier integration into spatial models. IDW is the most commonly used deterministic model in spatial interpolation [33,44,45]. Spatial interpolation methods assume a stronger correlation between nearby points than between more distant ones, a principle known as Tobler’s First Law of Geography [46]. IDW assumes that each measured point has a local influence that decreases with distance, meaning that closer points have similar values and greater influence on the interpolation, while more distant points are independent and have less influence [47]. In our study, the accumulated abundance values of the aquatic bird community and aquatic bird families at each counting point were used for interpolation.

Depth gradient data for the lake were provided by Autoridad Nacional del Agua (ANA), which conducted a bathymetric study, including a digital elevation model (DEM) [48]. To estimate chlorophyll-a content in Lake Piuray, we used the I543 index on Sentinel-2 satellite images, following the methodology described by Guigou [49]. Two study periods corresponding to the wet and dry seasons between 2022 and 2023 were selected. The images were processed using Google Earth Engine [50], where a customized script was implemented for data extraction and analysis. Image selection considered cloud-free periods for both study years. Before applying the I543 index, atmospheric and radiometric corrections were made to ensure accurate chlorophyll-a estimation.

To analyze the relationship between the distribution of the aquatic bird community and families and the physical variables of the lake (depth and chlorophyll-a), the ‘raster’ package [51] was used. The rasters generated by the IDW interpolation method were individually stacked with the DEM of Lake Piuray and the chlorophyll-a content raster. This resulted in a raster with a spatial resolution of 10 meters per pixel in each case. The GAM was then applied using the ‘mgcv’ package [52], chosen for its ability to capture complex nonlinear relationships between the response and explanatory variables without imposing parametric constraints [53]. A quasi-Poisson distribution with a logarithmic link function was used to address overdispersion in the data, and the Restricted Maximum Likelihood (REML) method was employed to fit the model. This approach allowed for flexible modeling of aquatic bird responses to environmental gradients, considering potential interactions between habitat structure (depth) and food availability (chlorophyll-a). It also enabled the generation of non-linear relationship plots and adjusted distribution maps for the entire community as well as for each aquatic bird family. The significance of each explanatory variable in the model was assessed with a threshold of P < 0.05. Additionally, the “gam.check” function in the ‘mgcv’ package was used to analyze and, if necessary, adjust the smoothing parameter (k) for each explanatory variable based on significance values. To determine the depth and chlorophyll-a intervals with the highest concentration of aquatic birds, both at the community and family levels, we used the adjusted density values obtained from the modeled rasters. Both environmental variables were divided into regular intervals: one-meter intervals for depth and 0.01-unit intervals (I543 index) for chlorophyll-a. Each raster cell was assigned to its corresponding interval, and the mean of the adjusted values within each class was calculated using the “dplyr” package [54] in R. The results were then represented graphically using bar plots, which allowed for the identification of specific depth and chlorophyll-a ranges associated with higher mean densities of aquatic birds.

### Analysis of seasonal and zonal influence on the aquatic bird community and families

At the community level, species richness, diversity (Shannon-Wiener index), and total abundance (measured as the maximum cumulative count) were calculated for each season and in each of the four study zones. To evaluate differences between seasons and zones regarding species richness, diversity, and community abundance, as well as the abundance of aquatic bird families, the Kruskal-Wallis test was used, followed by post-hoc comparisons using the Dunn test with Bonferroni correction. These analyses were performed using the ‘FSA’ package [55] in RStudio, considering a significance threshold of P < 0.05.

## Results

### Abundance patterns of the aquatic bird community and families

**Fig 2:**
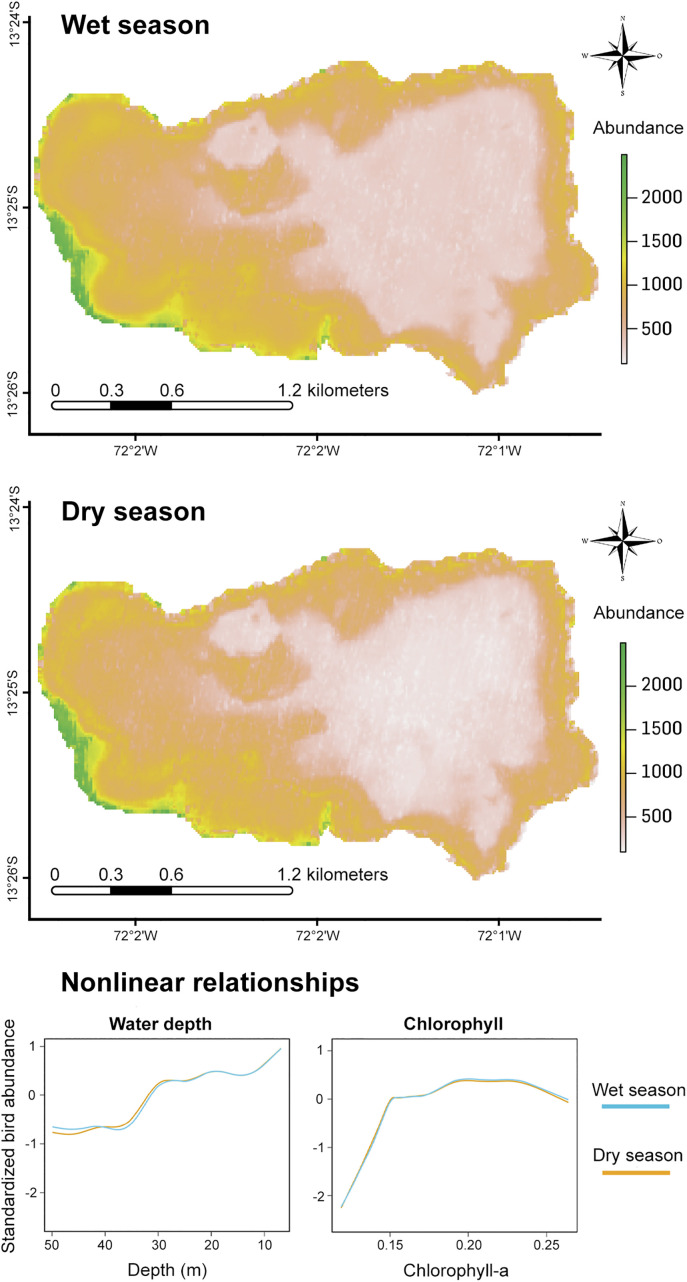
Distribution maps of the abundance of aquatic birds in Lake Piuray, estimated using a generalized additive model for the wet and dry seasons (above). The green shades represent areas with the highest concentration of individuals. Graphs of nonlinear relationships between environmental variables (water depth and chlorophyll-a concentration) and the abundance of aquatic birds in both seasons (below). Trend lines below zero on the Y-axis indicate negative relationships. The base map incorporates modified Copernicus Sentinel data [2022], sourced from Sentinel-2 MSI Level-1C imagery. Imagery is provided courtesy of the Copernicus Programme of the European Space Agency (ESA). Data are available via the Copernicus Data Space Ecosystem: https://link.dataspace.copernicus.eu/7n28. Use is permitted under the Copernicus Data License, which grants free, full, and open access under EU law, allowing reproduction, distribution, adaptation, and commercial use. For license details, visit: https://cds.climate.copernicus.eu/licences/ec-sentinel.

At the community level, the distribution maps obtained using the GAM model (Fig 2), based on IDW extrapolation, lake depth, and chlorophyll-a, showed similar explained deviance in both seasons: 44.1% for the wet season and 41.9% for the dry season. These maps reveal patterns with a considerably high abundance in Z1 and the westernmost part of Z2, both in the wet and dry seasons, corresponding to the southeastern end of the lake. On the other hand, the lowest values were recorded in the central part of the eastern half, coinciding with the deepest areas of the lake (Z3 and Z4). The nonlinear relationships for the depth variable (Fig 2) indicate an inverse relationship between aquatic bird abundance and depth in both seasons, with a decrease in abundance at depths greater than 30 m and higher concentrations at depths less than 3 m. Regarding chlorophyll-a, the highest abundance values were recorded between 0.20 and 0.25 (chlorophyll index I543), while negative relationships were observed at values below 0.15.

**Fig 3:**
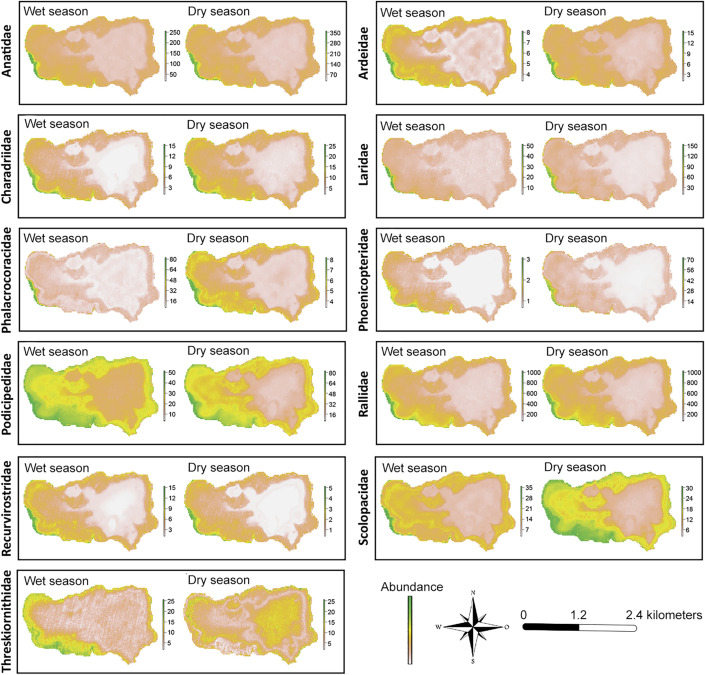
Distribution maps of the abundance of 11 aquatic bird families in Lake Piuray, estimated using a generalized additive model for the wet and dry seasons. Areas shaded in green represent zones with the highest concentration of individuals. The base map incorporates modified Copernicus Sentinel data [2022], sourced from Sentinel-2 MSI Level-1C imagery. Imagery is provided courtesy of the Copernicus Programme of the European Space Agency (ESA). Data are available via the Copernicus Data Space Ecosystem: https://link.dataspace.copernicus.eu/7n28. Use is permitted under the Copernicus Data License, which grants free, full, and open access under EU law, allowing reproduction, distribution, adaptation, and commercial use. For license details, visit: https://cds.climate.copernicus.eu/licences/ec-sentinel.

**Fig 4:**
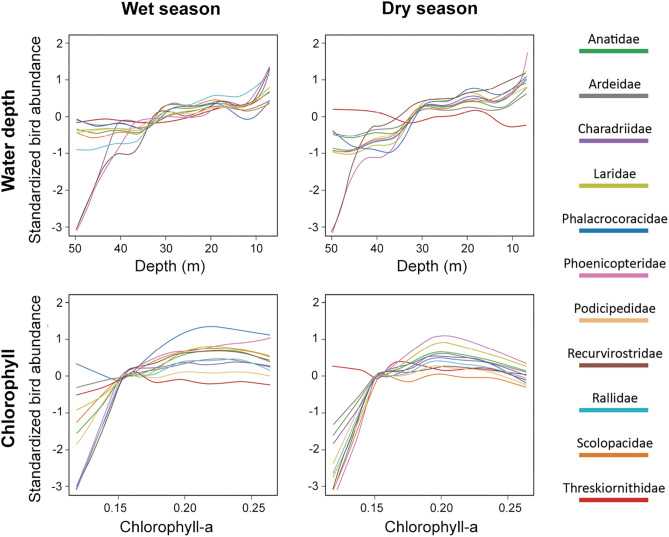
Nonlinear relationships between environmental variables (water depth and chlorophyll-a concentration) and the abundance of 11 aquatic bird families during the wet (left) and dry (right) seasons. The results were obtained using a generalized additive model. Each colored line in the graph represents an aquatic bird family, as shown in the legend. Trend lines below zero on the Y-axis indicate negative relationships, while lines above zero indicate positive relationships.https://doi.org/10.5281/zenodo.14902710.

At the family level, the GAM model performed similarly for 9 out of the 11 families studied, with an average explained deviance of 35.9% (± 9.1%) in the wet season and 38.1% (± 4.4%) in the dry season (Table 1). However, the families Ardeidae and Threskiornithidae showed significantly low values in both seasons. The adjusted distribution maps (Fig 3) reflected similar patterns for most families, with high concentrations between Z1 and the western end of Z2 and low concentrations in the deeper areas of the lake. However, the Threskiornithidae family showed an irregular distribution during the dry season. The families Charadriidae, Phoenicopteridae, and Recurvirostridae showed a more pronounced tendency for abundance in shallow waters (< 1 m) compared to other families (S4 File). In contrast, the Threskiornithidae family showed a relatively constant response in both seasons, suggesting a lower influence of depth on its abundance. Regarding chlorophyll-a, most families showed the highest abundance around 0.20 (I543 index), with negative relationships at values below 0.15, forming unimodal curves skewed toward areas with higher chlorophyll-a concentrations. However, the Threskiornithidae family exhibited a different response compared to the other families, as it did not show a defined peak or subsequent decline, indicating lower sensitivity to this variable compared to the other families Fig 4.

**Table 1:** Model fit metrics for aquatic bird abundance across seasons.

	Wet season		Dry season	
Family	Adjusted R²	Explained Deviance (%)	Adjusted R²	Explained Deviance (%)
Anatidae	0.394	38.9	0.333	36.4
Ardeidae	0.159	15.1	0.282	28.4
Charadriidae	0.368	45	0.331	40.8
Laridae	0.345	33.4	0.272	30.4
Phalacrocoracidae	0.238	20.8	0.376	40.7
Phoenicopteridae	0.309	44.1	0.328	42
Podicipedidae	0.32	30.7	0.328	32.5
Recurvirostridae	0.366	41.8	0.381	39.1
Rallidae	0.43	44.5	0.366	44
Scolopacidae	0.244	24.3	0.378	37.1
Threskiornithidae	0.153	13.3	0.0363	4.08

Adjusted coefficient of determination (adjusted R²) and percentage of deviance explained by the generalized additive model (GAM) for the abundance of aquatic birds in 11 families during the wet and dry seasons. The values reflect the model's fit and explanatory power in relation to the environmental variables considered.https://doi.org/10.5281/zenodo.14902748

### Influence of seasons and study zones on the aquatic bird community and families

**Fig 5:**
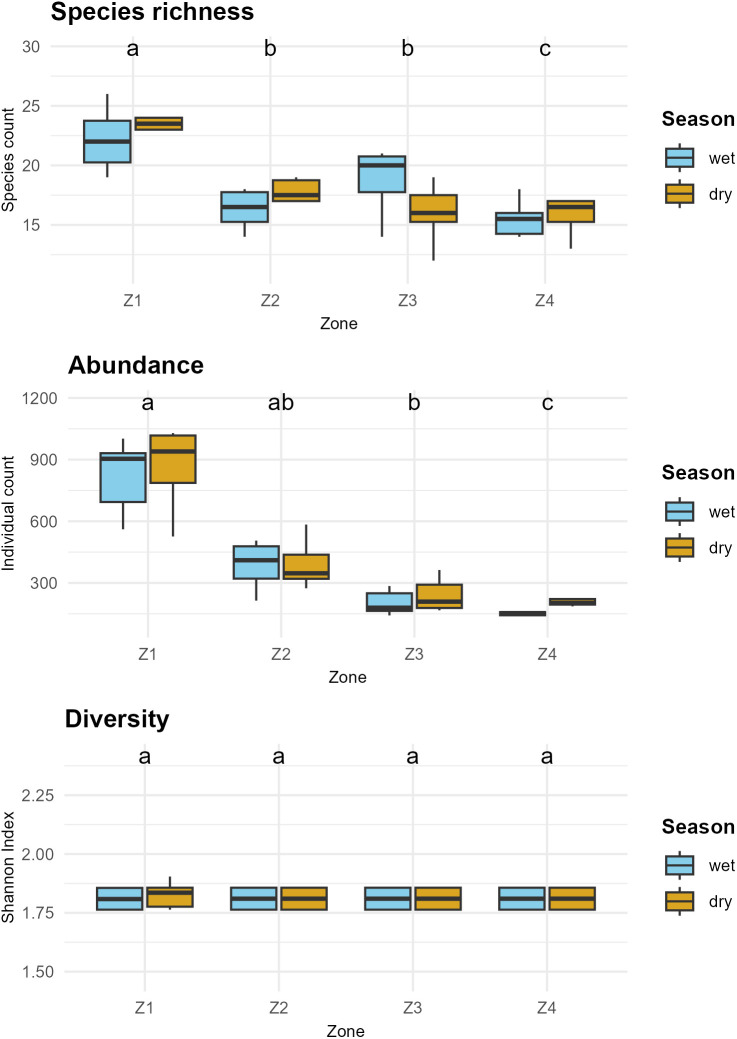
Comparison of species richness, abundance, and the Shannon diversity index between zones and seasons. The letters above the boxplots indicate significant differences between zones, as determined by the Dunn test with Bonferroni correction (p < 0.05). The colors represent the wet season (sky blue) and the dry season (dark yellow). https://doi.org/10.5281/zenodo.14902717.

A total of 44 species of aquatic birds, distributed across 11 families, were recorded, with a count of 19,768 individuals over 12 monthly censuses conducted at 13 counting points and 4 transects used for the avian community analysis. Statistical analysis showed no significant differences in species richness, diversity, and aquatic bird abundance between the wet and dry seasons (P > 0.05). However, when evaluating the influence of different zones, significant differences were identified in species richness and abundance among the four studied zones (P < 0.01), although no differences were found in diversity (P = 0.9965). Post hoc analyses using Dunn’s test revealed significant comparisons (adjusted P < 0.01), indicating that Z1 had the highest species richness (37 species) and abundance (10,148 aquatic birds), in contrast to Z4, which showed the lowest species richness (24 species) and abundance (2,303 aquatic birds) (Fig 5).

**Fig 6:**
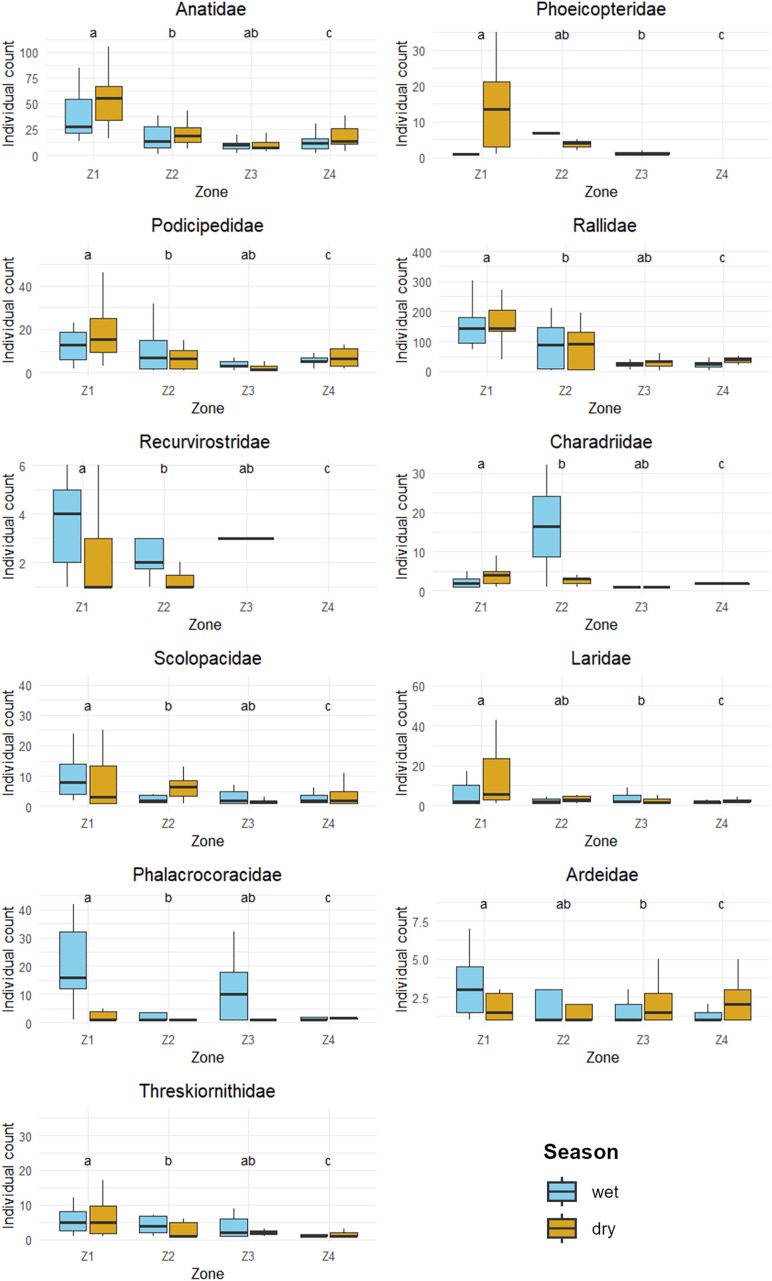
Comparison of the abundance of 11 families of aquatic birds across zones (Z1 to Z4) and seasons (wet and dry). The letters above the plots indicate significant differences between zones, as determined by the Dunn test with Bonferroni correction (p < 0.05). The colors represent the wet season (sky blue) and the dry season (dark yellow). https://doi.org/10.5281/zenodo.14902726.

The family-level analysis revealed significant differences between the wet and dry seasons in 6 of the 11 analyzed families (P < 0.05). Specifically, the families Phalacrocoracidae, Scolopacidae, and Recurvirostridae exhibited higher abundance during the wet season, while the families Charadriidae, Laridae, and Phoenicopteridae were more abundant in the dry season. The remaining five families (Anatidae, Podicipedidae, Rallidae, Ardeidae, and Threskiornithidae) showed no significant differences between seasons (Fig 6). The comparison between zones for each family revealed highly significant differences in 9 of the 11 families (P < 0.01), except for Ardeidae and Threskiornithidae, which exhibited marginal significance (P = 0.04611) and non-significance (P = 0.07295), respectively. Post hoc analysis indicated that, for most families, Z1 showed significant differences compared to the other zones (adjusted P < 0.05), with higher abundance. In contrast, no significant differences were found in any comparisons for the Ardeidae and Threskiornithidae families (adjusted P > 0.05).

## Discussion

This study represents the first analysis of the relationship between environmental factors and the distribution patterns of aquatic birds at two ecological scales (community and family levels) for a wetland located in the southeastern Peruvian Andes. The results provide a valuable contribution to understanding the high-Andean wetlands of the region, which exhibit biological similarities due to their geographic proximity [56]. The richness of aquatic birds recorded in this study, with 44 species, is comparable to that documented in other wetlands of significant ecological importance in the central Andes, such as the Ramsar site of Lucre-Huacarpay, where previous research reported between 30 and 50 species [57,58]. Furthermore, this value is consistent with figures observed in larger lakes of the Andean region, such as Lake Junín, which hosts 55 species [59], or Lake Titicaca, with 45 species [60].

At the community level, the GAM model identified the highest concentrations of aquatic birds and positive nonlinear relationships in the shallowest areas of the lake (< 1 m) during both seasons. This pattern is consistent with previous findings in Andean lakes of Ecuador [24], in tropical coastal lakes in southeastern Brazil [61], and even in simulated scenarios modeling aquatic bird abundance as a function of depth [11]. These results support the importance of depth as a determining factor in the distribution and abundance of aquatic birds [62,63]. Regarding primary production, represented by chlorophyll-a, the model showed that the highest concentrations of aquatic birds were associated with I543 index values between 0.20 and 0.26, corresponding to areas of higher productivity, consistent with other findings in wetlands of the eastern high Andes of Bolivia [64]. On the other hand, negative nonlinear relationships were observed at values below 0.15 in both seasons, reaffirming the strong correlation between this variable and the distribution and abundance of aquatic birds [65,66]. Although Lake Piuray exhibits an initial mesotrophic state, characterized by a moderate amount of nutrients [67], the relationship between primary productivity and aquatic birds suggests that even moderate nutrient levels can significantly influence the dynamics of these communities.

At the family level, distribution patterns exhibited various trends, as factors such as depth can elicit differentiated responses among families depending on depth gradients [68]. For instance, shorebird families like Charadriidae and Recurvirostridae showed nonlinear relationships with peaks in abundance only in very shallow waters and highly negative responses in deeper waters, as these aquatic birds prefer extremely shallow waters (< 0.10 m) [69]. Similarly, the Phoenicopteridae family, represented solely by *Phoenicopterus chilensis* in our study, preferred shallow waters with high primary productivity, a characteristic trait of this aquatic bird family [70]. Families with some diving species, such as Anatidae and Podicipedidae, showed greater tolerance in moderately deep waters (20–30 m), as evidenced by distribution maps that place a significant number of individuals from these families in areas with such depths. This aligns with findings by Colwell and Taft [71], who describe a higher number of diving species in seasonal wetlands in the southern California valley. A different case was the Threskiornithidae family, composed of *Plegadis ridgwayi* and *Theristicus branickii*, which showed non-linear relationships close to zero for both depth and chlorophyll-a content across both seasons. Additionally, this family had the lowest explained deviation values of all, with 13.3% in the wet season and 4.08% in the dry season (Table 1). This suggests that depth and chlorophyll-a content are not variables that explain the distribution and abundance of this aquatic bird family, which is consistent with observations during monthly surveys, where the highest number of individuals from this family was seen in agricultural fields surrounding the lake, regardless of the water depth near the fields. The Ardeidae family also showed a low explained deviation, especially in the wet season (15.1%). The results for these last two families may be explained by the strong association that ibises and herons have with vegetation abundance rather than with other environmental variables [19].

Regarding temporal variations at the community level, no significant differences were found in richness, abundance, and diversity between the wet and dry seasons. This result is consistent with previous research conducted in wetlands of the central and southern Andes of Peru [5,72] and could be explained by the high presence of resident species, including predominant ones. On the other hand, spatial variations were observed according to the zonation at the community level. Zone Z1 stood out as the area with the highest aquatic bird richness and abundance, associated with the largest extent of lakeshore beaches compared to the other zones. This factor has also been key in explaining aquatic bird richness and abundance in other Andean lakes, such as Lake Tota in the Eastern Cordillera of the Colombian Andes [73]. However, diversity remained similar across all studied zones, possibly due to the predominance of species like *Fulica ardesiaca*, which maintains abundant populations in these ecosystems [24,74].

At the family level, significant variations in the abundance of six aquatic bird families were identified between the dry and wet seasons. During the rainiest months of the wet season (December to February), the Phalacrocoracidae family, represented exclusively by *Phalacrocorax brasilianus*, and the Recurvirostridae family, dominated by *Himantopus mexicanus*, reached their peak abundances. This pattern is consistent with data from the eBird portal, which reports higher concentrations of these species in high-Andean lakes during these months [75]. On the other hand, the Scolopacidae family, comprising 11 species classified as boreal migrants [76], showed higher abundance during the wet season, although some individuals remained in the region during the dry season. This permanence could be explained by oversummering strategies, as reported in some shorebirds on the central coast of Peru [77]. During the dry season, the Charadriidae and Laridae families exhibited different abundance patterns: Charadriidae, with four species but dominated by *Vanellus resplendens*, and Laridae, with two species but predominantly *Chroicocephalus serranus*, reached their highest abundance levels in the driest months (June to August), although both families maintained considerable populations throughout the year. Notably, *V.resplendens* was reported to exhibit population movements from the headwaters of the basin toward Lake Piuray during the driest months [28]. Additionally, *C. serranus* is known to form flocks in the southern Andes of Peru during the dry [78]. For the Phoenicopteridae family, *P. chilensis* was the only representative and was observed only during the dry season and in November, with no records in other periods of the year. This constitutes one of the first documented records of its wandering movements in Lake Piuray. The changes in the population of these six aquatic bird families, alternating between the dry and wet seasons, could be one of the reasons why no significant difference was found in the aquatic bird community between the two seasons. Notable differences were also observed among the different study zones, particularly in Zone Z1, which hosted the highest abundance for most families, except for the Ardeidae and Threskiornithidae families, which showed no significant differences between zones. Zone Z1 was characterized by having the largest areas of shallow waters and lakeshore beaches, factors closely related to high richness and abundance of aquatic birds [79]. In contrast, Zone Z4 had the largest extent of deep waters and an almost nonexistent lakeshore beach, in addition to being close to a rural road and a eucalyptus forest, which likely explains the low levels of richness and abundance in this zone.

The conservation of Andean wetlands requires spatially precise predictions that consider both natural and anthropogenic aspects to identify the most important sites for conservation efforts [80]. In this study, the distribution patterns of aquatic birds appear to result from the interplay between natural and semi-natural habitat factors present in all study zones. The GAM models, which analyzed the influence of depth and chlorophyll-a concentration on aquatic birds, explained between 35% and 44% of the observed variability in the distribution of these species at the community and family levels, with depth being the most influential variable in all cases. These results suggest that other factors may also play an important role in the distribution of aquatic birds in Lake Piuray. These could include local environmental factors, such as the presence or absence of emergent vegetation, which influences species that use it as refuge [81], or the influence of dominant generalist species, which can displace less abundant species [24], as is the case with *Fulica ardesiaca*, which has a large population in the Lake Piuray [[28]]. Additionally, other factors such as wetland size, which in most study cases shows a positive correlation with species richness [82], water chemistry parameters like pH, which is associated with certain aquatic bird guilds [83], or urban development, which tends to reduce aquatic bird richness in wetlands [84], could also be determinants. Therefore, it is essential to include a greater number of variables in future studies to gain a more comprehensive understanding of the factors explaining the distribution of aquatic birds in these ecosystems.

## Conclusions

Our findings contribute to identifying priority areas for the management and conservation of aquatic birds in Lake Piuray, a representative area of the humid puna wetlands. We found that the shallowest areas of the lake (< 1 m depth) with intermediate-to-high chlorophyll-a content (I543: 0.20–0.25) host the highest abundance and species richness of aquatic birds at both community and family levels. Consistent with global patterns, shorebird families (e.g., Charadriidae, Recurvirostridae) exhibited a strong affinity for very shallow waters (< 1 m), a trait particularly critical in this high-Andean system where littoral zones are scarce and disproportionately impacted by human activity. In contrast, diving birds (e.g., Anatidae, Podicipedidae) displayed broader tolerance to deeper waters (up to 30 m). Notably, the Threskiornithidae family showed no clear association with depth or chlorophyll-a, suggesting reliance on alternative habitat features such as adjacent agricultural fields. Overall, our observations underscore that shallow, productive zones, though limited in area, are vital for aquatic bird conservation in high Andean wetlands, warranting targeted protection amid growing anthropogenic pressures.

## Supporting information

S1 File List of aquatic bird species and their abundance by season in Lake Piuray. List according to the South American Classification Committee (SACC) of 2025.
(XLSX)

S2 File Bird abundance data at the community and family levels for creating maps using IDW interpolation. (XLSX)

S3 File Raster data of chlorophyll-a for the wet and dry seasons in Lake Piuray. The base map incorporates modified Copernicus Sentinel data [2022], sourced from Sentinel-2 MSI Level-1C imagery. Imagery is provided courtesy of the Copernicus Programme of the European Space Agency (ESA). Data are available via the Copernicus Data Space Ecosystem: https://link.dataspace.copernicus.eu/7n28. Use is permitted under the Copernicus Data License, which grants free, full, and open access under EU law, allowing reproduction, distribution, adaptation, and commercial use. For license details, visit: https://cds.climate.copernicus.eu/licences/ec-sentinel.
(ZIP)

S4 File Abundance values according to depth and chlorophyll interval. (PDF)

S5 File Data for analyzing the influence of seasons and zones on the community and families of aquatic birds in Lake Piuray. (XLSX)

S6 File Bird count database, organized by zone on a monthly basis. (XLSX)

S1 Table Results of the Kruskal-Wallis and Dunn test for the comparison of aquatic bird families across seasons. (PDF)

S2 Table Results of the Kruskal-Wallis and Dunn test for the comparison between zones by families. (PDF)
