# Supplementary material for: Distribution patterns of aquatic birds in a high-Andean wetland in southeastern Peru: An approach based on environmental factors
Source: PLoS One. 2026 Mar 26;21(3):e0320987. doi: 10.1371/journal.pone.0320987 (PMC13020964; doi:10.1371/journal.pone.0320987)

Average abundance of the waterbirds community by depth interval

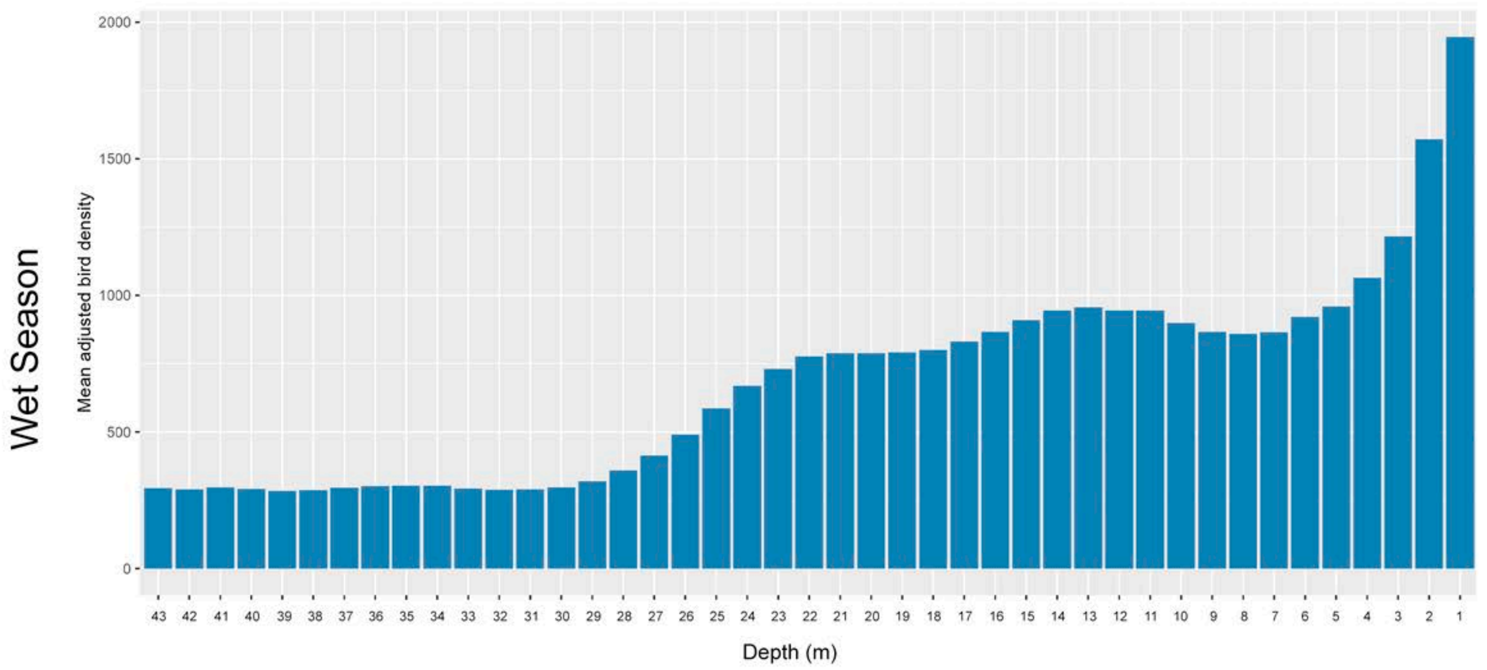

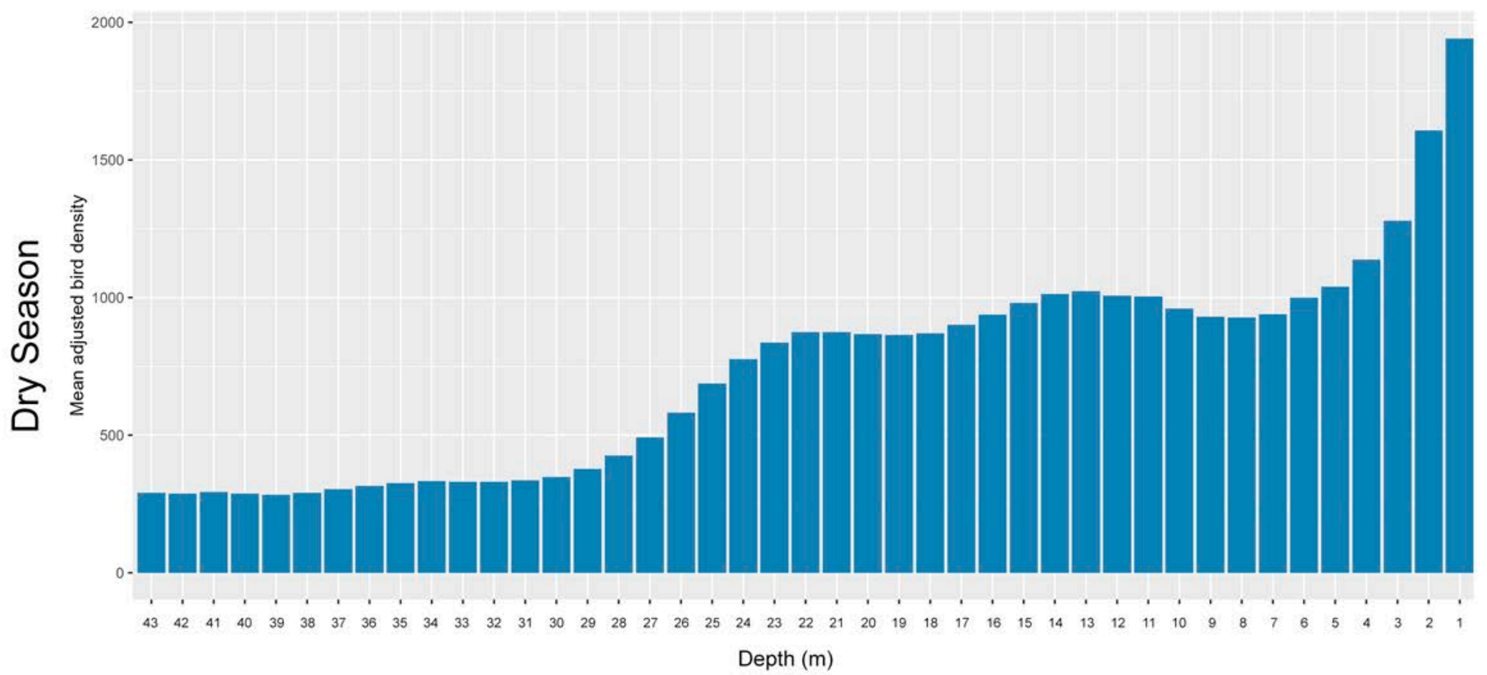

Average abundance of waterbirds families by depth

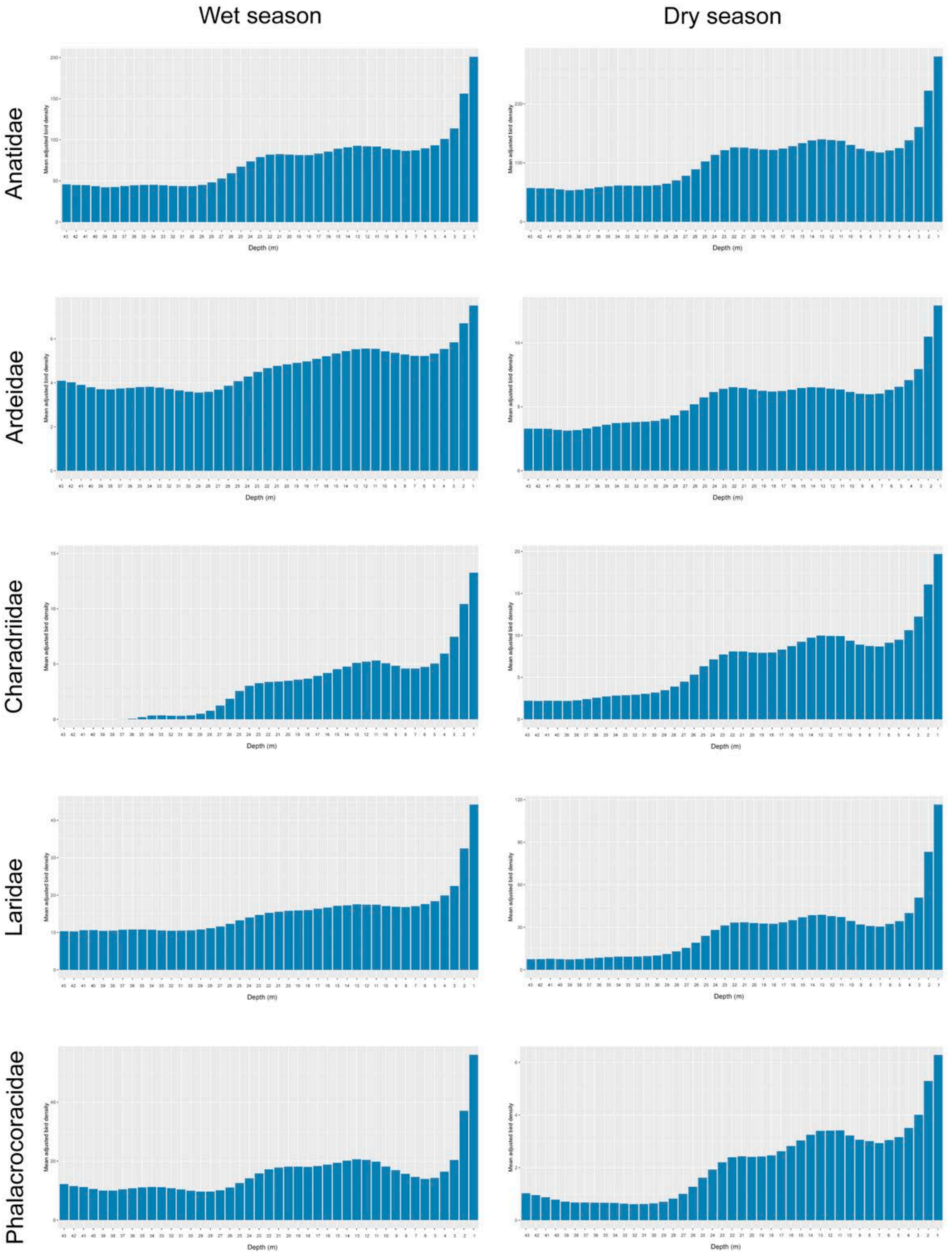

Average abundance of waterbirds families by depth interval

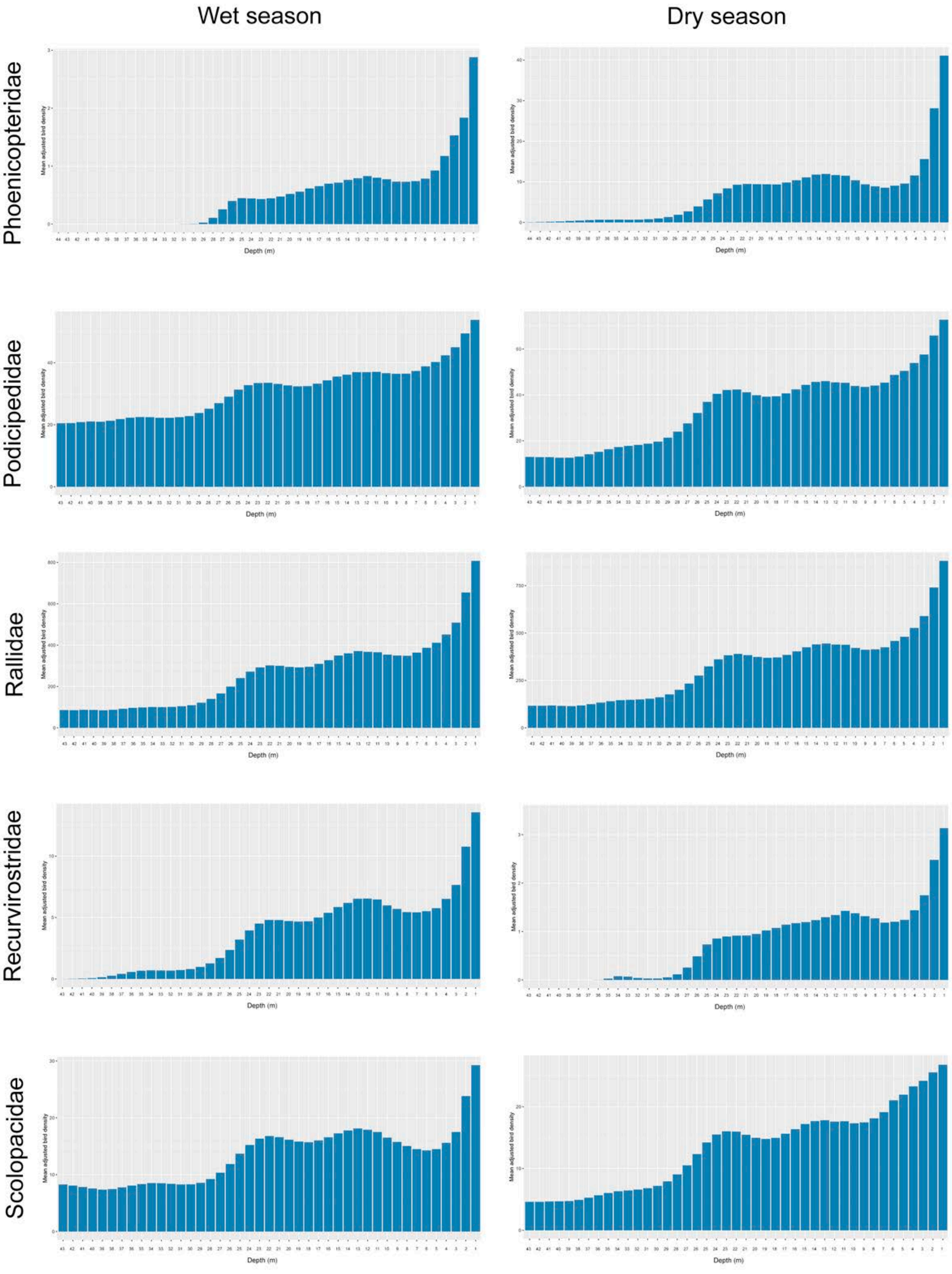

Average abundance of waterbirds families by depth interval

Threskiornithidae

Wet season

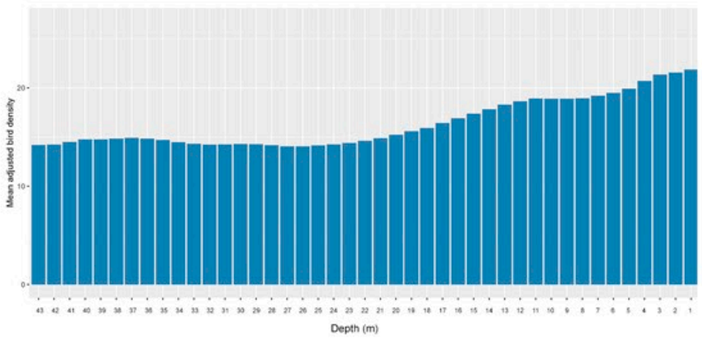

Dry season

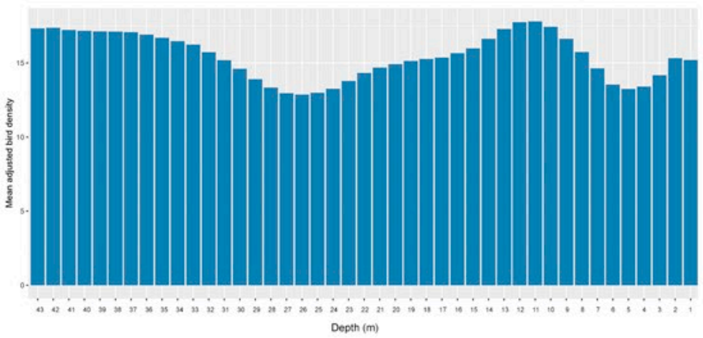

Average abundance of the waterbirds community by depth interval

Wet Season

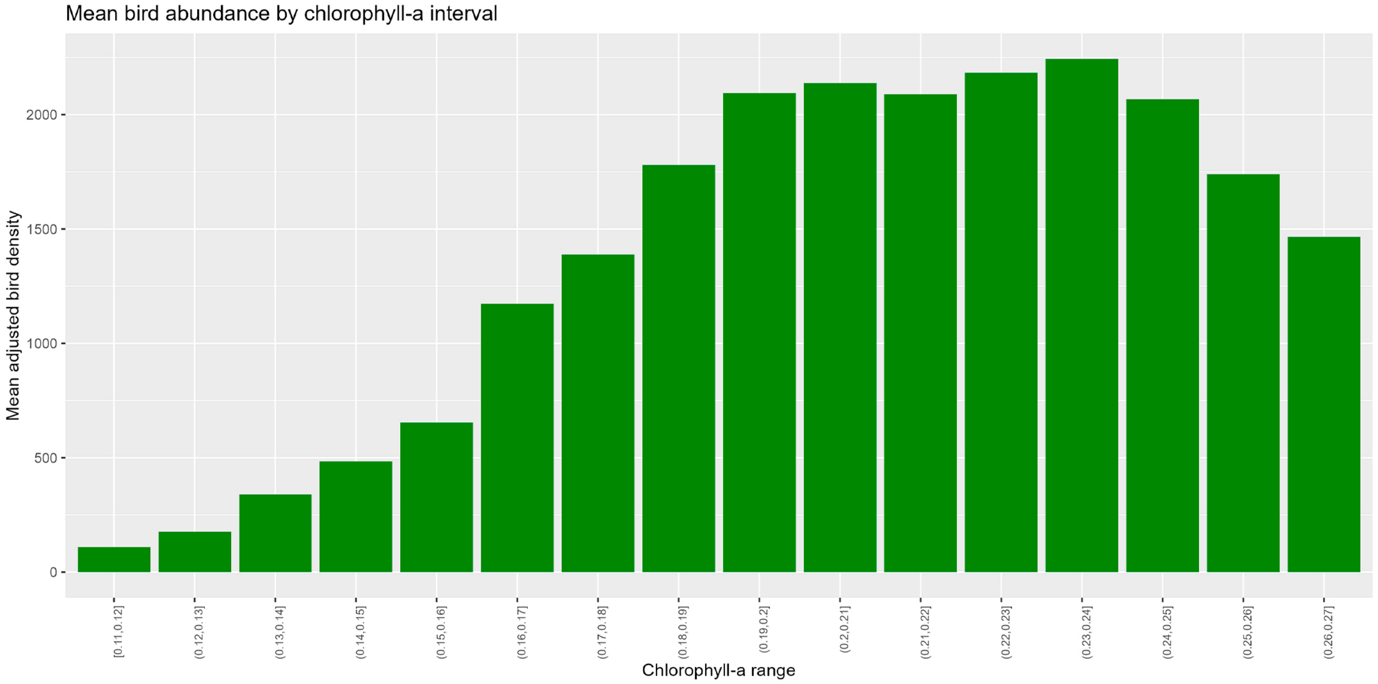

Dry Season

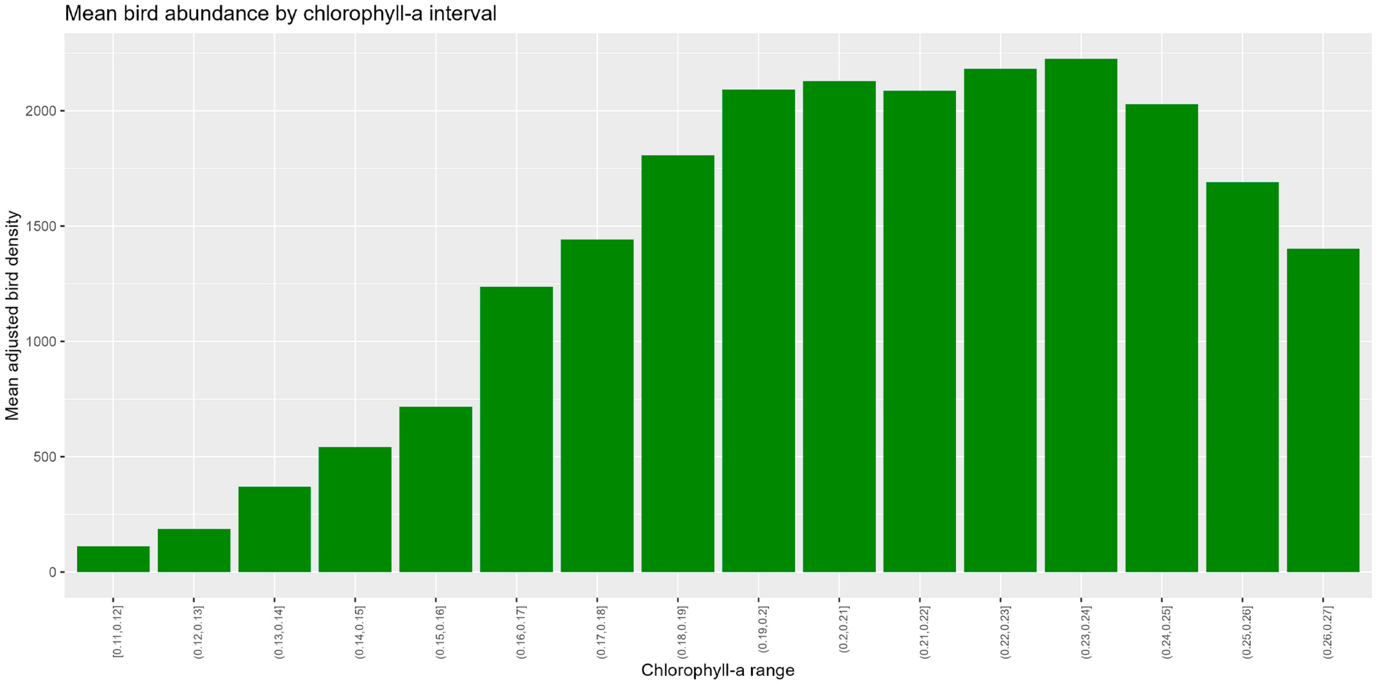

Average abundance of waterbirds families by depth interval

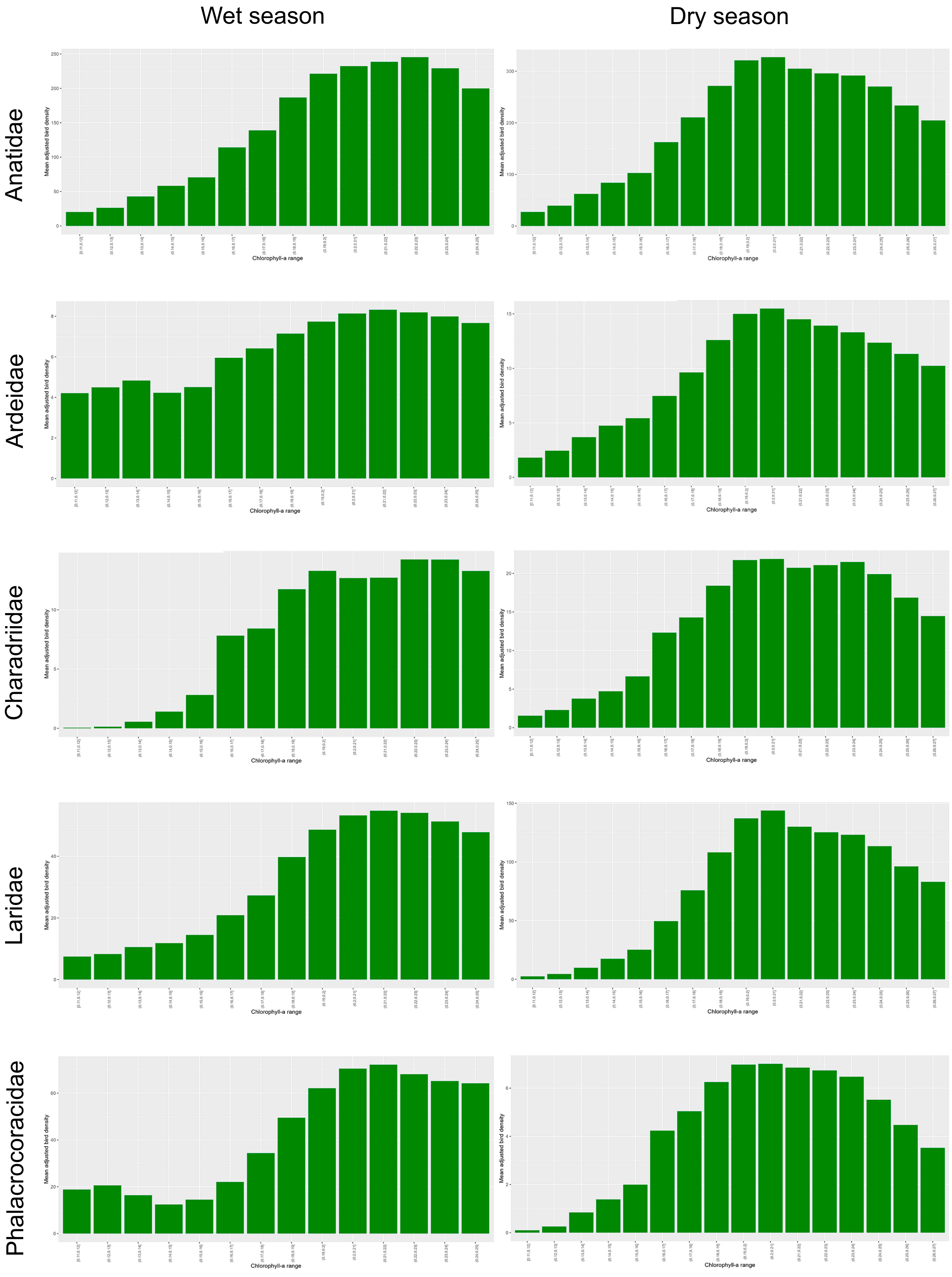

Average abundance of waterbirds families by depth interval

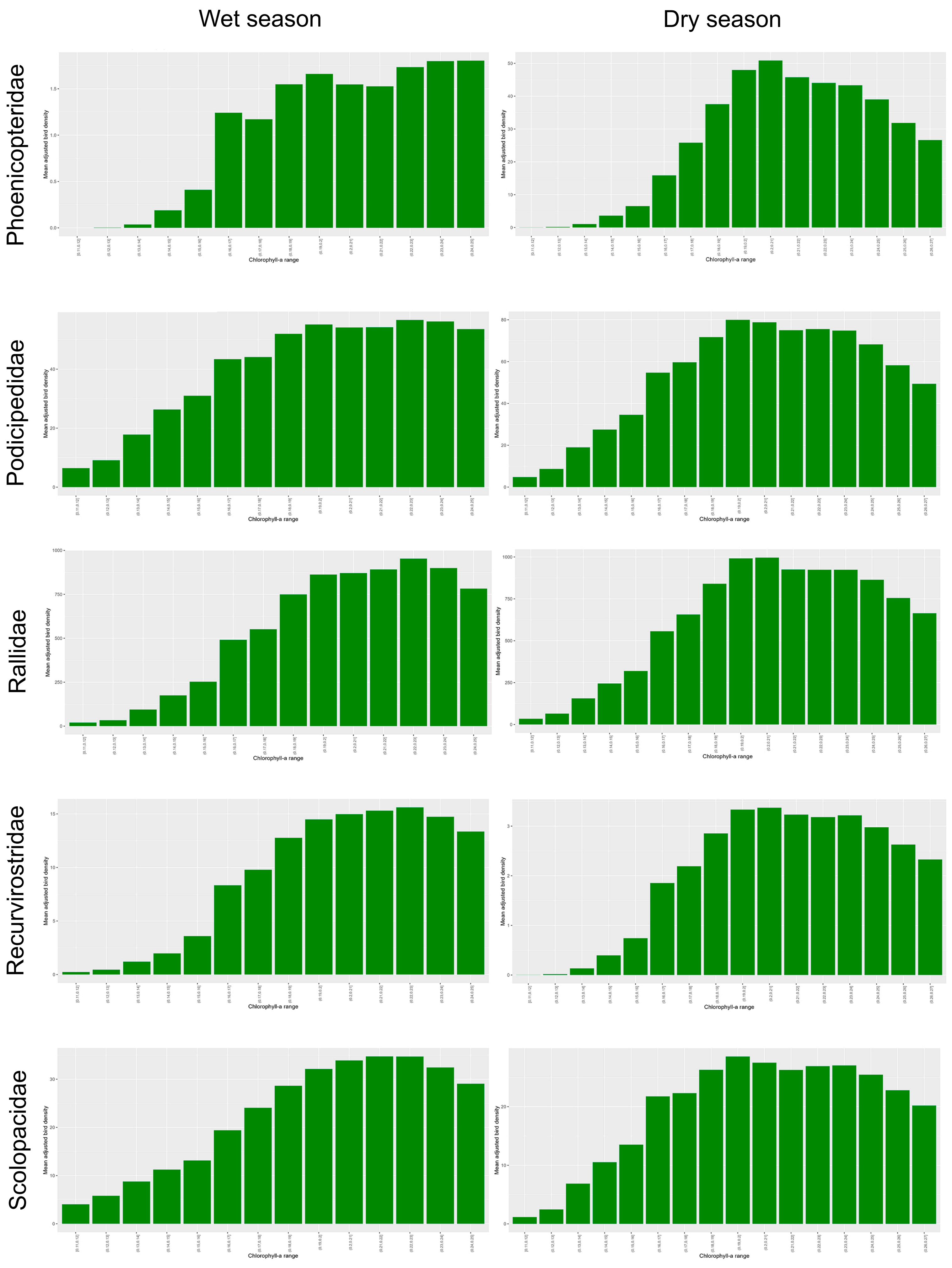

Average abundance of waterbirds families by depth interval

Wet season

Dry season

Threskiornithidae

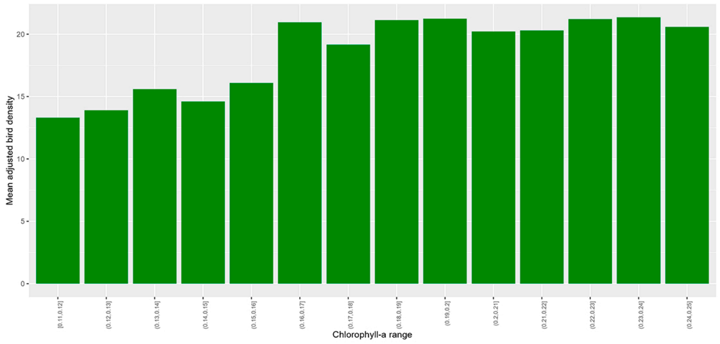

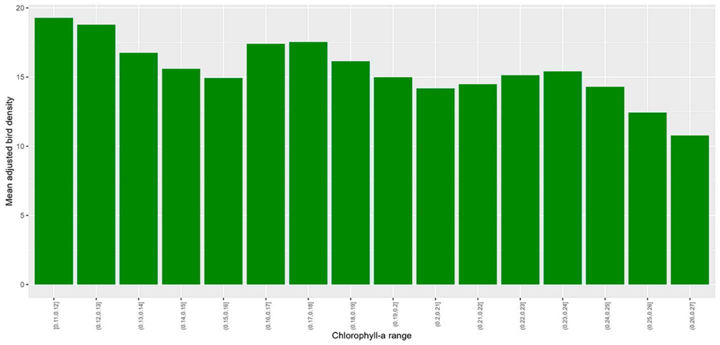

Supplement: S4 File — (PDF) [file pone.0320987.s004.pdf]
