# Supplementary material for: Distribution patterns of aquatic birds in a high-Andean wetland in southeastern Peru: An approach based on environmental factors
Source: PLoS One. 2026 Mar 26;21(3):e0320987. doi: 10.1371/journal.pone.0320987 (PMC13020964; doi:10.1371/journal.pone.0320987)
Supplement: S1 Table — (PDF) [file pone.0320987.s007.pdf]

Results of the Kruskal-Wallis and Dunn test for the comparison of aquatic bird families across seasons.

The table presents the chi-square statistic, degrees of freedom (*df*), and *p*-value obtained from the Kruskal-Wallis test, followed by the post hoc Dunn test for significant comparisons. The *Z* value, unadjusted (*P. unadj*), and adjusted (*P. adj*) *p*-values are reported for pairwise comparisons between the wet and dry seasons. Statistically significant comparisons ($p < 0.05$) indicate differences in the abundance or distribution of bird families between seasons.

Family	Chi-square	df	p-valor	Significant comparison	Z	P. unadj	P. adj
Anatidae	2.5888	1	0.1076	Wet - Dry	-1.608987	0.107619	0.107619
Ardeidae	0.040158	1	0.8412	Wet - Dry	0.2003936	0.841173	0.841173
Charadriidae	11.768	1	0.000603	Wet - Dry	-3.430468	0.000603	**0.000603**
Laridae	7.4355	1	0.006395	Wet - Dry	-2.726806	0.006395	**0.006395**
Phalacrocoracidae	7.9293	1	0.004864	Wet - Dry	2.815901	0.004864	**0.004864**
Phoeicopteridae	8.3402	1	0.003878	Wet - Dry	-2.887938	0.003878	**0.003878**
Podicipedidae	0.231	1	0.6308	Wet - Dry	0.4806271	0.630782	0.630782
Rallidae	1.515	1	0.2184	Wet - Dry	-1.230848	0.218380	0.218380
Recurvirostridae	14.065	1	0.000177	Wet - Dry	3.750372	0.000177	**0.000177**
Scolopacidae	10.294	1	0.001335	Wet - Dry	3.208422	0.001335	**0.001335**
Threskiornithidae	0.74776	1	0.3872	Wet - Dry	0.8647322	0.387186	0.387186
