# Supplementary material for: Distribution patterns of aquatic birds in a high-Andean wetland in southeastern Peru: An approach based on environmental factors
Source: PLoS One. 2026 Mar 26;21(3):e0320987. doi: 10.1371/journal.pone.0320987 (PMC13020964; doi:10.1371/journal.pone.0320987)
Supplement: S2 Table — (PDF) [file pone.0320987.s008.pdf]

Results of the Kruskal-Wallis and Dunn test for the comparison between zones by families.

Results of the Kruskal-Wallis and Dunn test for the comparison between zones by waterbird families. The table presents the chi-square statistic (*Chi-square*), degrees of freedom (*df*), and *p*-value from the Kruskal-Wallis test. For significant post hoc comparisons, the *Z* values, unadjusted *p*-values (*p-value*), and adjusted *p*-values (*P adj*) from the Dunn test are provided. Statistically significant comparisons ($p < 0.05$) indicate differences in the distribution of waterbird families across zones.

Family	Chi-square	df	*p*-value	Significant comparison	Z	*p*-value	*P* adj
Anatidae	58.624	3	1.157e-12	Z1 - Z3	7.219259	5.227e-13	3.136e-12
				Z1 - Z4	5.668843	1.438e-08	8.626e-08
				Z1 - Z2	5.049807	4.423e-07	2.654e-06
Ardeidae	7.9955	3	0.04611	Z1 - Z2	2.580744	9.859e-03	5.915e-02
Charadriidae	53.609	3	1.36e-11	Z1 - Z4	6.597857	4.171e-11	2.503e-10
				Z1 - Z3	5.988976	2.112e-09	1.267e-08
				Z1 - Z2	4.730292	2.242e-06	1.345e-05
Laridae	16.612	3	0.0008492	Z1 - Z4	3.725832	1.947e-04	1.168e-03
				Z1 - Z3	3.242721	1.184e-03	7.104e-03
Phalacrocoracidae	12.202	3	0.006723	Z1 - Z2	2.922713	3.470e-03	2.082e-02
Phoeicopteridae	14.511	3	0.002286	Z1 - Z4	3.685830	2.280e-04	1.368e-03
				Z1 - Z3	2.669831	7.589e-03	4.553e-02
Podicipedidae	53.359	3	1.537e-11	Z1 - Z3	7.299795	2.882e-13	1.729e-12
				Z1 - Z4	3.721409	1.981e-04	1.189e-03
				Z2 - Z3	3.418371	6.300e-04	3.780e-03
				Z3 - Z4	-3.578386	3.457e-04	2.074e-03
Rallidae	61.591	3	2.687e-13	Z1 - Z4	6.182810	6.297e-10	3.778e-09
				Z1 - Z3	7.265084	3.728e-13	2.237e-12
				Z1 - Z2	4.179331	2.924e-05	1.754e-04
				Z2 - Z3	3.085753	2.030e-03	1.218e-02
Recurvirostridae	50.246	3	7.083e-11	Z1 - Z4	6.241583	4.332e-10	2.599e-09
				Z1 - Z3	5.960636	2.513e-09	1.508e-08
				Z1 - Z2	3.470077	5.203e-04	3.122e-03
				Z2 - Z4	2.771506	5.580e-03	3.348e-02
Scolopacidae	26.701	3	6.802e-06	Z1 - Z2	4.602172	4.181e-06	2.509e-05

				Z1 - Z3	4.292673	1.765e-05	1.059e-04
				Z1 - Z4	3.358420	7.839e-04	4.703e-03
Threskiornithidae	6.9672	3	0.07295	Z1 - Z4	2.568709	1.021e-02	6.125e-02
